# Feasibility and accuracy evaluation of three human papillomavirus assays for FTA card-based sampling: a pilot study in cervical cancer screening

**DOI:** 10.1186/s12885-015-1882-9

**Published:** 2015-11-04

**Authors:** Shao-Ming Wang, Shang-Ying Hu, Wen Chen, Feng Chen, Fang-Hui Zhao, Wei He, Xin-Ming Ma, Yu-Qing Zhang, Jian Wang, Priya Sivasubramaniam, You-Lin Qiao

**Affiliations:** 1 Cancer Institute/Hospital, Chinese Academy of Medical Sciences and Peking Union Medical College, P.O. Box 2258, 17 South Panjiayuan Lane, Beijing, 100021 China; 2 Beijing Municipal Institute of Labour Protection, Beijing, 100021 China; 3 Vanderbilt University School of Medicine, Nashville, TN 37212 USA

**Keywords:** Whatman Indicating FTA Elute® card (FTA card), Cervical cancer screening, *care*HPV™, Cobas®4800, Hybrid capture 2 (HC2)

## Abstract

**Background:**

Liquid-state specimen carriers are inadequate for sample transportation in large-scale screening projects in low-resource settings, which necessitates the exploration of novel non-hazardous solid-state alternatives. Studies investigating the feasibility and accuracy of a solid-state human papillomavirus (HPV) sampling medium in combination with different down-stream HPV DNA assays for cervical cancer screening are needed.

**Methods:**

We collected two cervical specimens from 396 women, aged 25–65 years, who were enrolled in a cervical cancer screening trial. One sample was stored using DCM preservative solution and the other was applied to a Whatman Indicating FTA Elute® card (FTA card). All specimens were processed using three HPV testing methods, including Hybrid capture 2 (HC2), *care*HPV™, and Cobas®4800 tests. All the women underwent a rigorous colposcopic evaluation that included using a microbiopsy protocol.

**Results:**

Compared to the liquid-based carrier, the FTA card demonstrated comparable sensitivity for detecting high grade Cervical Intraepithelial Neoplasia (CIN) using HC2 (91.7 %), *care*HPV™ (83.3 %), and Cobas®4800 (91.7 %) tests. Moreover, the FTA card showed a higher specificity compared to a liquid-based carrier for HC2 (79.5 % vs. 71.6 %, *P* = 0.015), comparable specificity for *care*HPV™ (78.1 % vs. 73.0 %, *P* > 0.05), but lower specificity for the Cobas®4800 test (62.4 % vs. 69.9 %, *P* = 0.032). Generally, the FTA card-based sampling medium’s accuracy was comparable with that of liquid-based medium for the three HPV testing assays.

**Conclusions:**

FTA cards are a promising sample carrier for cervical cancer screening. With further optimization, it can be utilized for HPV testing in areas of varying economic development.

## Background

Cervical Cancer is the fourth most common cancer of women worldwide. An estimated 528,000 new cases and 266,000 deaths from cervical cancer occur annually, among which 85 % occur in developing countries. China, a developing country with high cervical cancer incidence (7.5/100,000) and mortality (3.4/100,000), bears a substantial burden of cervical disease [1, 2].

Current evidence shows that persistent infection with carcinogenic human papillomavirus (HPV) types is the cause of virtually all cervical cancer [3]. Consequently, HPV DNA testing was explored as one of the primary cervical cancer screening methods in several countries [4]. In developing countries like China, where critical infrastructure is not available, screening by gynecologists and cytologists is generally inefficient and unworkable. Expanding the coverage of objective HPV testing methods is a more expeditious and effective way to introduce large-scale of cervical cancer screening. Combining cervical sample collection with regional or centralized HPV detection could be an efficient way to increase screening coverage in such areas. However, most current HPV testing methods utilize a liquid-based sample carrier, which is limited by large sample volumes, risk of leakage, and challenges with storage and transportation, especially in remote rural areas.

Solid-state specimen carriers, such as the Whatman Indicating FTA Elute® card (FTA card, GE Healthcare, Buckinghamshire, UK), provide an alternative sampling medium for DNA sample collection. FTA cards provide a cost effective room temperature method for collecting, shipping, archiving and processing nucleic acids from a wide variety of biological samples. The cards contain an inert dye that changes from purple to white indicating the location of a clear, colorless sample. Moreover, indicating FTA Elute cards facilitate rapid purification of nucleic acids in less than 30 min per sample and provide DNA in solution for multiple amplification reactions with high stability. There is evidence that genomic DNA stored on FTA cards at room temperature for more than 17.5 years still can be successfully amplified by PCR. FTA cards have been used in forensic science for DNA sample collection and storage for decades, but it has only recently been explored as an alternative to current liquid-based sampling mediums for cervical cancer screening [5–9].

A few studies have investigated the FTA cards with PCR-based detection methods or a single HPV DNA testing method with high clinical sensitivity and specificity [5, 9–12]. However, no published study has made a direct comparison of FTA cards and liquid sampling medium using the predominant clinical HPV testing methods.

Therefore, our project sought to investigate the feasibility of using FTA cards as an HPV sampling medium for cervical cancer screening. We also explored the compatibility of FTA cards with the predominant clinical HPV DNA assays.

## Methods

### Study population

Samples were collected among women enrolled in “LCMCCSS”, a large population-based screening trial in rural China, which was a collaboration between the Cancer Institute, Chinese Academy of Medical Sciences (CICAMS) and the Program for Appropriate Technology in Health (PATH) [13, 14]. Eligible women were ages 25–65 years, not pregnant and did not have a history of diagnosed Cervical Intraepithelial Neoplasia (CIN), cervical cancer, or hysterectomy. After a socio-demographic questionnaire survey, each woman provided one self-collected and two clinician-collected specimens. The self-collected specimen and one clinician-collected specimen were tested by *care*HPV™ and Hybrid Capture 2 (Qiagen, Gaithersburg, MD, USA); the other clinician-collected specimen was tested for HPV16/18/45 E6 protein with the OncoE6™ Cervical test (Arbor Vita Corporation, Sunnyvale, CA, USA). After sample collection, women were screened by visual inspection with acetic acid (VIA). One to two weeks later, women who tested positive for any of the six screening tests performed (VIA, HPV E6, and HC2 and *care*HPV™ on clinician-collected and self-collected specimens) and approximately a 10 % random sample of women who tested negative for all screening tests (screen-negative women) were called back to undergo a second VIA and a rigorous colposcopic evaluation. All colposcopically detected abnormalities (acetowhite lesions) were biopsied. If the colposcopic examination showed no lesion in a quadrant but any of the screen results was positive, a random biopsy was obtained at the squamocolumnar junction in that quadrant at 2, 4, 8, or 10 o’clock. An endocervical curettage (ECC) was performed after the cervical biopsies. Women who were screen negative and were without any colposcopic indications of abnormality did not undergo colposcopically-directed biopsies.

### Sample collection

**Fig. 1:**
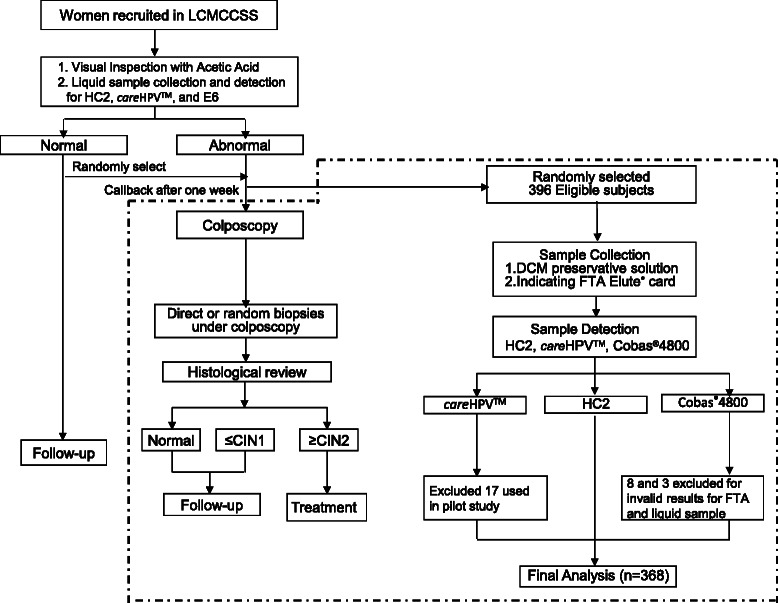
Study Flow Chart.Note: CIN1 cervical intraepithelial neoplasia grade 1, CIN2 cervical intraepithelial neoplasia grade 2, FTA Whatman Indicating FTA Elute® card, HC2 Hybrid capture 2

We conducted our study among women who returned for colposcopy in LCMCCSS between May and June 2012. Recruitment was stopped when the target sample size (*n* = 396) was reached. The population included 207 women who were HPV positive, 17 women who were VIA positive (including 10 HPV positive women) and 182 randomly selected normal women. Ethics approval was obtained from the Ethics Committee of Cancer Institute and Hospital, Chinese Academy of Medical Sciences (Approval No. 12-040/574). Written Informed consent was obtained from each subject. One liquid sample was collected by a physician using a Conical Cervical Sampler (Qiagen, Gaithersburg, MD, USA) and was stored in DCM preservative solution (Qiagen, Gaithersburg, MD, USA). A second sample was collected by a physician using a new Conical Cervical Sampler and applied to an FTA card, which will be referred to as the “solid sample” in this study. All the samples were stored at room temperature and analyzed in the CICAMS central laboratory. All the women underwent a rigorous colposcopic evaluation that included using a microbiopsy protocol as previously described [13, 15]. A CICAMS pathologist was responsible for reporting the results and high grade CIN case confirmation (see Fig. 1).

### Liquid sample detection

HC2, the first HPV DNA testing method approved by the United States Food and Drug Administration for clinical use, utilizes hybrid capture technology to detect 13 carcinogenic HPV genotypes. HC2 testing was conducted per the manufacturer’s instruction, with one exception: 50 μl of the DCM specimen was combined with 25 μl of the kit denaturation reagent rather than combining 1000 μl of STM specimen with 500 μl of the kit denaturation reagent. *care*HPV™ is a newly-developed and promising screening system developed for low resource regions. Its workflow is similar but simpler than the HC2 test with a lower cost and simpler administration. *care*HPV™ testing was conducted according to the manufacturer’s instructions as previously described [16]. A signal strength of 1.0 relative light units per positive control (rlu/pc) or greater was considered positive for both tests. The Cobas®4800 test (Roche Molecular Systems, Pleasanton, CA) is a widely-used, PCR-based testing method with high clinical sensitivity and specificity [17, 18]. It can detect 14 types of high-risk HPV and requires a lower number of DNA copies as compared to the HC2 test. Since the manufacturer did not provide instructions for DCM-based sample detection, CICAMS developed the workflow according to several small pilot studies. DCM preservative solution was diluted 10 times with sterilized PBS buffer, referring to *care*HPV™ which had approved commercial instructions for DCM samples. Thereafter, DNA extraction and PCR amplification were performed according to the manufacturer’s instructions.

### FTA card sample detection

In order to ensure a high degree of accuracy, CICAMS and GE Healthcare performed pilot tests to develop optimized protocols for use of FTA cards with the three HPV assays. FTA cards were punched using a specifically designed sterilized perforator (3-mm Harris Uni-Core device; Whatman, GE Healthcare, Buckinghamshire, UK). As HC2 and Cobas®4800 test used the same buffer, those tests shared nine punched disks while the *care*HPV™ assays used another six disks. This allowed us to obtain the required testing concentration for each of the HPV assays. The FTA card was chemically treated with proprietary reagents that lyse cells upon contact, causing the release of nucleic acids. DNA was recovered from the FTA elute matrix through a simplified elution process using heat and water. Inhibitory components, such as hemoglobin, were retained on the FTA elute matrix.

The six disks and nine disks were transferred into separate 1.5-ml microfuge tubes. Five hundred microliter of sterile water was added to each tube, and the tubes were immediately pulse vortexed for 15 s. Water was removed with a sterile fine-tip pipette. This process was performed twice. Sixty microliter of DCM preservative solution and 90 μl of DEPC water were added to each tube. Both tubes were centrifuged for 30 s and transferred to a heating block at 95 °C for 30 min. During the incubation period, both tubes were centrifuged for 30 s every 10 min to minimize condensation. At the end of heating process, both tubes were pulse vortexed approximately 30 s and centrifuged for 30 s. The eluted DNA was placed into new microcentrifuge tubes and stored at −80 °C until analysis. Finally, 50 μl of the eluate originating from the nine disks was used for HC2 and 25 μl was used for Cobas®4800. Similarly, 50 μl of the eluate from the six disks was used for the *care*HPV™ assay. Thereafter, PCR amplification was performed to acquire final results for Cobas®4800 test.

### Statistical analysis

The primary objective of this study was to evaluate the sensitivity and specificity of FTA card-based sampling for the detection of CIN2+. According to previous studies, we assumed that the sensitivity of detecting CIN2+ would range from 0.85 to 0.96, the specificity of detecting CIN2+ would range from 0.60 to 0.80, and the CIN2+ prevalence would be approximately 4 %. Given these parameters, we calculated a necessary sample size of 375 with an alpha of 0.05 and 80 % power [19]. Assuming a 5 % testing failure rate, a maximum of 395 women was required. Demographic information such as age, marital status, educational level, occupation, and sexual history was quantified. The accuracy of each combination of different sampling media and downstream HPV testing methods was presented as sensitivity, specificity, and positive and negative predictive values. Proportions were compared using the Fisher’s exact test. SAS 9.2 was used to analyze data (SAS Institute, Cary NC). The 95 % confidence interval (95 % CI) was calculated using the Wald test by OpenEpi Version 3 (www.OpenEpi.com), an open source epidemiologic statistics for public health. Statistical significance was assessed by two-tailed tests with an alpha level of 0.05.

## Results

### Demographic information

A total of 3191 women were invited to participate in screening through the LCMCCSS study in 2012, among which 370 (11.7 %) refused to participate. The participants had a higher marriage rate (96.7 % vs. 93.8 %, *P* = 0.005) and a lower smoking rate (0.3 % vs. 1.6 %, *P* = 0.003) than non-participants. Based on their test results, 1392 women were referred for colposcopy, among which 106 (7.6 %) refused to return. The women who returned for colposcopy (*n* = 1286) had a lower smoking rate (0.1 % vs. 1.9 %, *P* = 0.016) and fewer live births (<3 live births: 91.2 % vs. 84.9 %, *P* = 0.031) compared to those who refused to return. For this study, 396 women, with a mean age of 43.7 ± 8.9 years, were recruited from among the women who were referred for colposcopy. All of the subjects were married and the average ages of initiation of sexual activity and menarche were 20.2 ± 2.2 years and 15.5 ± 1.7 years, respectively. More than half of the women (56.5 %) reported primary school as their highest educational level, 33 % reported middle school, and only 10.5 % reported high school or college education. The majority were farmers or housewives (73.5 %), non-smokers (99.3 %), and non-drinkers (86.4 %). Ninety-seven percent (96.9 %) of the women reported having three or less sexual partners, 62.7 % had three or less pregnancies, and 85.4 % had three or fewer live births (Table 1).

**Table 1:** Demographic information of the participants

Variable	Value
Age (Mean ± SD)	43.7 ± 8.9
Age of sexual debut (Mean ± SD)	20.2 ± 2.2
Age of menarche (Mean ± SD)	15.5 ± 1.7
Marriage (YES, %)	100
Highest education level (%)	
Primary school	56.5
Middle school	33
High school and above	10.5
Occupation, (%)	
Farmer	39.8
Housewife	33.7
Other	26.5
Smoking (NO, %)	99.3
Drinking (NO, %)	86.4
Oral contraceptive (YES, %)	1.9
Sexual partner (*n* ≥ 3, %)	3.1
No. of pregnancies (%)	
≤ 3	62.7
> 3	37.3
No. of live births (%)	
≤ 3	85.4
> 3	14.6

*SD* standard deviation and standard error of the mean

### Pathological confirmation and valid cases

Of the 396 samples, 17 were used for *care*HPV™ pilot testing. In addition, eight FTA card samples and three DCM samples did not provide valid results for the Cobas® 4800 test. Therefore, our final analysis was conducted using 368 samples that had valid results for all three HPV tests. Specifically, 34 cases were diagnosed as CIN Grade 1 (CIN1), nine as CIN Grade 2 (CIN2), two as CIN Grade 3 (CIN3), and one case as High Grade Cervical Glandular Intraepithelial Neoplasia (HCGIN).

### HPV positivity and accuracy of detecting CIN2+ for different screening methods

VIA showed a high specificity (95.8 %) for detecting CIN2 or worse (CIN2+) lesions, but a low sensitivity (8.3 %). Only one of 12 CIN2+ cases was identified by VIA. HPV prevalence and the accuracy of detecting CIN2+ lesions for six combinations of two sampling mediums with three HPV testing methods are shown in Table 2. The liquid samples showed comparable HPV positivity among HC2 (30.4 %), *care*HPV™ (28.8 %) and Cobas®4800 (32.1 %) tests. Compared to the liquid samples, FTA-based samples showed lower HPV positivity for HC2 (22.8 %) and *care*HPV™ (23.9 %), but a higher HPV positivity for Cobas®4800 test (39.4 %). The FTA card evidenced higher specificity for the HC2 test compared to the liquid medium (79.5 % vs. 71.6 %, *P* = 0.015), but a relatively lower specificity for the Cobas®4800 test (62.4 % vs. 69.9 %, *P* = 0.032). For the *care*HPV™ test, two sampling methods demonstrated comparable specificity (FTA vs. Liquid: 78.1 % vs. 73.0 %, *P* > 0.05). For the other accuracy parameters, FTA and the conventional DCM sampling medium provided comparable results. Specifically, the FTA card demonstrated a sensitivity identical with that of the DCM medium for HC2 (91.7 %), *care*HPV™ (83.3 %), and Cobas®4800 test (91.7 %). Moreover, with similar negative predictive value (NPV), the FTA card showed comparable positive predictive values (PPV) for HC2 (FTA vs. Liquid: 13.1 % vs. 9.8 %, *P* = 0.47), *care*HPV™ (FTA vs. Liquid: 11.4 % vs. 9.4 %, *P* = 0.66), and Cobas®4800 (FTA vs. Liquid: 7.6 % vs. 9.3 %, *P* = 0.61) tests.

**Table 2:** Accuracy of detecting CIN2+ for different sampling media in combination with three HPV testing methods

Methods	Sampling Medium	HPV Prevalence	Accuracy							
		(%)	Sensitivity		Specificity		Positive Predictive Value		Negative Predictive Value	
			% (*n*/*N*)	95 % CI	% (*n*/*N*)	95 % CI	% (*n*/*N*)	95 % CI	% (*n*/*N*)	95 % CI
HC2	FTA	22.8	91.7 (11/12)	76.1 ~ 100.0	79.5 (283/356)	75.3 ~ 83.7	13.1 (11/84)	5.9 ~ 20.3	99.6 (283/284)	98.9 ~ 100.0
	Liquid	30.4	91.7 (11/12)	76.1 ~ 100.0	71.6 (255/356)	66.9 ~ 76.3	9.8 (11/112)	4.3 ~ 15.3	99.6 (255/256)	98.8 ~ 100.0
*care*HPV™	FTA	23.9	83.3 (10/12)	62.2 ~ 100.0	78.1 (278/356)	73.8 ~ 82.4	11.4 (10/88)	4.8 ~ 18	99.3 (278/280)	98.3 ~ 100.0
	Liquid	28.8	83.3 (10/12)	62.2 ~ 100.0	73.0 (260/356)	68.4 ~ 77.6	9.4 (10/106)	3.8 ~ 15	99.2 (260/262)	98.1 ~ 100.0
Cobas®4800	FTA	39.4	91.7 (11/12)	76.1 ~ 100.0	62.4 (222/356)	57.4 ~ 67.4	7.6 (11/145)	3.3 ~ 11.9	99.6 (222/223)	98.8 ~ 100.0
	Liquid	32.1	91.7 (11/12)	76.1 ~ 100.0	69.9 (249/356)	65.1 ~ 74.7	9.3 (11/118)	4.1 ~ 14.5	99.6 (249/250)	98.8 ~ 100.0

95 % *CI* 95 % confidence interval, *FTA* Whatman Indicating FTA Elute® card, *HC2* Hybrid capture 2

Detection results of individual analyses for 12 CIN2+ cases are shown in Table 3. The results showed that the HC2 and *care*HPV^TM^ tests failed to identify one and two CIN2+ cases, respectively. The Cobas®4800 test identified all the CIN2+ lesions except for one case of HCGIN. We also calculated the overall percent agreement of three HPV testing methods between two sampling mediums. The results showed a good overall percent agreement of 90.2 % (95%*CI*: 87.2–93.3 %), 83.7 % (95%*CI*: 79.9–87.5 %), and 85.6 % (95%*CI*: 82.0–89.2 %) between FTA card and liquid based sampling methods for HC2, *care*HPV™, and Cobas®4800 tests, respectively (Table 4).

**Table 3:** CIN2+ case detection by different combinations of HPV testing methods and sampling media

ID no.	Pathological result	HC2 (Rlu/PC)		*care*HPV™ (Rlu/PC)		Cobas® 4800	
		Liquid	FTA	Liquid	FTA	Liquid	FTA
447	CIN2	0.20^a^	181.73	0.42^a^	17.11	POS	POS
445	CIN2	99.90	22.42	51.31	28.50	POS	POS
444	CIN3	472.51	650.36	170.00	173.28	POS	POS
442	CIN2	675.40	325.26	117.93	140.85	POS	POS
448	CIN2	451.36	43.57	104.47	34.00	POS	POS
441	CIN2	2003.50	19.03	150.79	37.03	POS	POS
446	CIN2	4.26	0.28^a^	1.39	0.58^a^	POS	POS
443	CIN2	463.18	150.22	80.80	89.48	POS	POS
450	CIN2	1031.00	92.23	100.65	61.33	POS	POS
240	HCGIN	1.05	1.34	0.29^a^	0.78^a^	NEG^a^	NEG^a^
288	CIN3	26.62	1.42	20.65	3.71	POS	POS
344	CIN2	5.00	50.89	2.68	29.71	POS	POS

*CIN2* cervical intraepithelial neoplasia grade 2, *CIN3* cervical intraepithelial neoplasia grade 3, *HCGIN* high grade cervical glandular intraepithelial neoplasia, *FTA* Whatman Indicating FTA Elute® card, *HC2* Hybrid capture 2, *NEG* negative result, *POS* positive result, *RLU*/*PC* the ratio of relative light units to standard positive control, cut-point: 1.0

^a^CIN2+ case failed to be detected

**Table 4:** Performance and discrepancies between two specimen storage methods for three HPV testing assays

Test assays	FTA aample	Liquid sample		Subtotal	Agreement		
					%	95 % CI	
HC2		Positive	Negative		90.2	87.2	93.3
	Positive	80	4	84			
	Negative	32	252	284			
	Subtotal	112	256	368			
careHPV™		Positive	Negative		83.7	79.9	87.5
	Positive	67	21	88			
	Negative	39	241	280			
	Subtotal	106	262	368			
Cobas4800		Positive	Negative		85.6	82.0	89.2
	Positive	105	40	145			
	Negative	13	210	223			
	Subtotal	118	250	368			

## Discussion

According to our results, compared to current liquid-based HC2 testing, using FTA cards significantly increases test specificity while maintaining comparable sensitivity. These test characteristics could greatly reduce the referral rate for colposcopic examinations. Moreover, FTA cards provided comparable accuracy compared to the liquid-based medium for *care*HPV™, thereby providing an alternative sampling method for cervical cancer screening in remote areas. For the Cobas®4800 tests, FTA card-based samples showed a higher HPV positivity and a lower specificity of detecting CIN2+ compared to liquid samples. Further optimization of the workflow for the Cobas®4800 test is required to reduce false positives.

The combination of FTA cards with HC2 tests was previously explored in Holland [10]. Our study demonstrated a better overall percent agreement (China vs. Holland: 90.2 % vs. 77.4 %) between FTA cards and the liquid medium and a higher sensitivity for CIN2+ detection (91.7 % vs. 53.8 %) compared to the Dutch study. Different workflows, sample sizes and sample utilization may explain this discrepancy. In this study, FTA card-based samples were expected to be eluted to HPV concentrations comparable to those from DCM preservative solution for the HC2 and *care*HPV™ assays. However, concentration discrepancies remained when transferring samples from solid to liquid, which resulted in a relatively lower HPV positivity and a higher specificity for FTA card-based samples. *care*HPV™ demonstrated a relatively lower sensitivity than that of HC2, both in our study (*care*HPV™ vs. HC2: 83.3 % vs. 91.7 %) and in previous studies (*care*HPV™ vs. HC2: 90.0 % vs. 96.3 %) [16, 20]. However, considering the simplicity of sample transportation and storage, lower costs, and simpler administration, it is nonetheless a promising tool for future large scale cervical cancer screening projects, particularly in low resource settings.

Good agreement between liquid-based media and FTA cards was previously reported for several PCR-based methods, including GP5+/6+, SPF(10) PCR/DEIA /LiPA(25), and real-time PCR assays [7, 8, 12]. However, limited studies have investigated the accuracy of the combination of FTA cards and PCR-based methods for CIN2+ detection. A Spanish study showed that the combination of FTA cards and GP5+/6+ or SPF(10) HPV testing provided a sensitivity of 95.9–98.0 % and a specificity of 42.9–48.1 % for CIN2+ detection [11].

Our study is the first to explore the accuracy of FTA cards in combination with the Cobas®4800 test for CIN2+ detection. As the manufacturer did not provide instructions for DCM-based sample detection, CICAMS developed the workflow based on several small pilot studies. Specifically, samples used for PCR amplification from FTA cards had a much higher HPV concentration compared to samples from DCM preservative solution (3.6 % vs. 0.5 %), resulting in a high sensitivity (91.7 %). As a result, a higher HPV positivity and a lower specificity were found for FTA card-based samples compared to DCM samples. Further study is needed to optimize the workflow for Cobas®4800 testing using FTA card-based samples in order to reduce false positives and improve the specificity. Moreover, although this result was comparable with previous studies (70.97 %) of Cobas®4800 test in China [21] and other PCR-based methods (42.9–48.1 %) [11], one needs to take into consideration additional triage biomarkers in order to reduce the referral rate for colposcopy. Furthermore, Cobas®4800 testing identified all of the squamous epithelial lesions but missed one glandular epithelial lesion (one case of HCGIN). This reflects the general deficiency of HPV DNA based screening methods, and need for complementary cytology.

This is the first report of a study comparing three downstream HPV testing methods with clinical implications by FTA cards and conventional liquid based sampling media. This study explored experimental workflows for the *care*HPV™ and Cobas®4800 tests and optimized the current workflow for the HC2 test, which provides data for future laboratory investigations. Moreover, this study differs from previous reports which used PreservCyt (Hologic, Bedford, MA) medium for a single test; this study is the first to compare the accuracy of the FTA card and DCM preservative solution combined with three clinical HPV DNA tests in cervical cancer screening. This study has some limitations that must be addressed as well. First, our study was based on a referral population that was triaged for colposcopy. A higher HPV prevalence was found in this group compared to the general population, which had an impact on the accuracy parameters for detecting CIN2+ lesions. Second, this study is a hospital-based pilot study with a relatively small sample size. We therefore lacked statistical power to assess test characteristics among sub-groups. Third, the Cobas®4800 test workflow requires further optimization for future large-scale studies. Fourth, we did not consider the sequence of sample collection. Taking two samples with a short time interval might have a negative impact on the accuracy of the second sample. However, the second sample, which was collected on FTA cards, showed comparable accuracy for detecting CIN2+. This indicates that FTA cards have a robust capacity for capturing samples. Future studies should further evaluate the accuracy of these two sampling media in a random sampling sequence to avoid any potential bias. Fifth, we made a parallel comparison for three HPV testing methods on a single FTA card, which may result in insufficient distribution of sample for each test. Additionally, reproducibility studies were not feasible in this study. Finally, there are several types of cervical cytological sampling brushes, but this study only used a conical cervical sampler for sample collection. Future comparisons of different cytological samplers need to be investigated to identify the samplers that provide the highest degree of accuracy.

This work also draws a promising picture for future large-scale cervical cancer screening projects. As dried material on a solid carrier is neither hazardous nor contagious, applying genital self-samples on FTA cards can solve storage and transportation problems encountered in developing regions. With further optimization and automation of the experimental procedures, FTA cards in combination with *care*HPV™ could be used for cervical cancer screening in remote areas, and FTA cards in combination with HC2 or Cobas®4800 could be used in metropolitan areas. Our previous study demonstrated that the agreement between self-collected and clinician-collected specimens on FTA cards was very good and the acceptability of FTA card-based self-collection was reasonably high [5, 6]. Therefore, future studies should focus on a feasibility and accuracy evaluation of FTA-card based self-sampling in cervical cancer screening. It is possible that, in the future, women will be able to self-sample at home, and mail the sample to the regional central laboratory for sample analysis. Doctors would only call back those with positive result for colposcopic examination. Consequently, limited healthcare resources could be focused on high-risk populations, thereby increasing the coverage of current screening initiatives. Additionally, previous studies demonstrated acceptable performance of FTA cards in humid tropical climates with self-collection, which also provides this solid sample carrier a promising future in cervical cancer screening in low-resource areas [8, 22].

## Conclusions

Our study provided clinical evidence for evaluating the accuracy of FTA card-based sampling as an alternative to the liquid-based sampling in cervical cancer screening. With further optimization of the experimental workflow, FTA cards can be paired with different HPV DNA testing methods suitable a variety of economic conditions, thereby providing new possibilities for large-scale population-based cervical cancer screening.

## Abbreviations

CICAMS: Cancer Institute, Chinese Academy of Medical Sciences
CIN: Cervical Intraepithelial Neoplasia
CIN1: CIN Grade 1
CIN2: CIN Grade 2
CIN2+: CIN2 or worse
CIN3: CIN Grade 3
FTA card: Whatman Indicating FTA Elute® card
HC2: Hybrid Capture 2
HCGIN: High Grade Cervical Glandular Intraepithelial Neoplasia
HPV: Human Papillomavirus
NPV: Negative predictive value
PATH: Program for Appropriate Technology in Health
PPV: Positive Predictive Value
VIA: Visual Inspection with Acetic Acid
